# Pharmacokinetics in Mouse and Comparative Effects of Frondosides in Pancreatic Cancer

**DOI:** 10.3390/md14060115

**Published:** 2016-06-17

**Authors:** Jasem Al Shemaili, Khatija A. Parekh, Robert A. Newman, Björn Hellman, Carl Woodward, Abdu Adem, Peter Collin, Thomas E. Adrian

**Affiliations:** 1 Department of Physiology, Faculty of Medicine, United Arab Emirates University, P.O. Box 17666, Al Ain, UAE; 201617836@uaeu.ac.ae (J.A.S.); kparekh@uaeu.ac.ae (K.A.P.); 2 Phoenix Biotechnology Inc., San Antonio, TX 78217, USA; rnewman@phoenixbiotechnology.com; 3 Department of Pharmaceutical Biosciences, Uppsala University, Uppsala 75105, Sweden; Bjorn.Hellman@farmbio.uu.se; 4 Coastside Bio Resources, Deer Isle, ME 04627, USA; carlw@coastsidebio.com (C.W.); petercollin@coastsidebio.com (P.C.); 5 Department of Pharmacology, Faculty of Medicine, United Arab Emirates University, P.O. Box 17666, Al Ain, UAE; abdu.adem@uaeu.ac.ae

**Keywords:** frondoside A, pancreatic cancer, cancer, pharmacokinetics

## Abstract

The frondosides are triterpenoid glycosides from the Atlantic sea cucumber *Cucumaria frondosa*. Frondoside A inhibits growth, invasion, metastases and angiogenesis and induces apoptosis in diverse cancer types, including pancreatic cancer. We compared the growth inhibitory effects of three frondosides and their aglycone and related this to the pharmocokinetics and route of administration. Frondoside A potently inhibited growth of pancreatic cancer cells with an EC_50_ of ~1 µM. Frondoside B was less potent (EC_50_ ~2.5 µM). Frondoside C and the aglycone had no effect. At 100 µg/kg, frondoside A administered to CD_2_F_1_ mice as an *i.v.* bolus, the Cp_max_ was 129 nM, Cl_tb_ was 6.35 mL/min/m^2^, and half-life was 510 min. With *i.p.* administration the Cp_max_ was 18.3 nM, Cl_tb_ was 127 mL/min/m^2^ and half-life was 840 min. Oral dosing was ineffective. Frondoside A (100 µg/kg/day i.p.) markedly inhibited growth cancer xenografts in nude mice. The same dose delivered by oral gavage had no effect. No evidence of acute toxicity was seen with frondoside A. Frondoside A is more potent inhibitor of cancer growth than other frondosides. The glycoside component is essential for bioactivity. Frondoside A is only effective when administered systemically. Based on the current and previous studies, frondoside A appears safe and may be valuable in the treatment of cancer.

## 1. Introduction

Pancreatic cancer is a disease with a dismal prognosis and little hope for cure because effective therapies are not available [1]. While the disease is not very common it is a leading cause of cancer death because of the poor prognosis and the difficulty of detecting the disease at an early stage [2]. The mean survival time after diagnosis is about five months and even the subgroup (less than 20% of patients), in whom potentially curative surgery is an option, rarely survive more than two or three years [2]. Until recently, gemcitabine was the mainstay of therapy for pancreatic cancer patients in neoadjuvant, adjuvant and palliative treatment protocols [3]. While this drug improved quality of life, it has proved to have little effect on survival [3]. New developments in treatment strategies include the use of FOLFIRINOX and Nab-paclitaxel, both of which have shown some survival benefits over gemcitabine alone and only time will reveal their overall impact on the disease. However, new therapeutic strategies are urgently required for pancreatic cancer patients [3].

The frondosides are triterpenoid glycosides isolated from the Atlantic sea cucumber, *Cucumaria frondosa*. Frondoside A has potent antiproliferative, anti-invasive and anti-angiogenic effects on a variety of cancers including adenocarcinomas of the pancreas, breast, lung, and prostate as well as in leukemia [4,5,6,7,8]. Frondoside A has been shown to block cancer cell growth and apoptosis both *in vitro*, as well as in human cancer xenografts in athymic mice [4,5,6,7]. Frondoside A has also been shown to have synergistic effect when combined with gemcitabine *in vitro* as well as in human cancer xenografts in athymic mice [9]. Additionally, frondoside A has been shown to potently enhance innate immunity in laboratory animals [10], and to show immunomodulatory effects in splenocytes by proteomic analysis [11].

One aim of the present study was to compare the growth inhibitory effects of frondoside A to the other frondosides (B and C), and the aglyocone compound. These were isolated from the same sea cucumber species—*Cucumaria frondosa*. Additional aims included investigation of the pharmacokinetics of frondoside A following either *i.v.*, *i.p.*, or oral administration.

## 2. Results

### 2.1. Effects on Proliferation of AsPC-1 and S2013 Pancreatic Cancer Cells in Vitro

**Figure 1:**
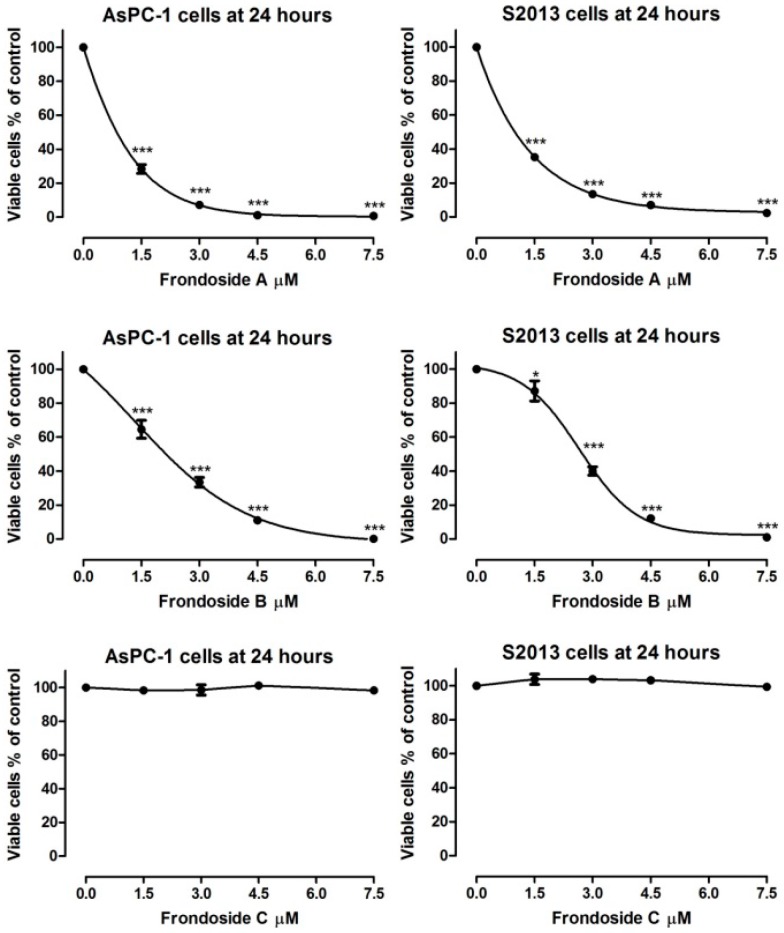
Effects of frondoside A, B and C on viability of AsPC-1 and S2013 human pancreatic cancer cells after 24 h of incubation. *** *p* < 0.001.

**Figure 2:**
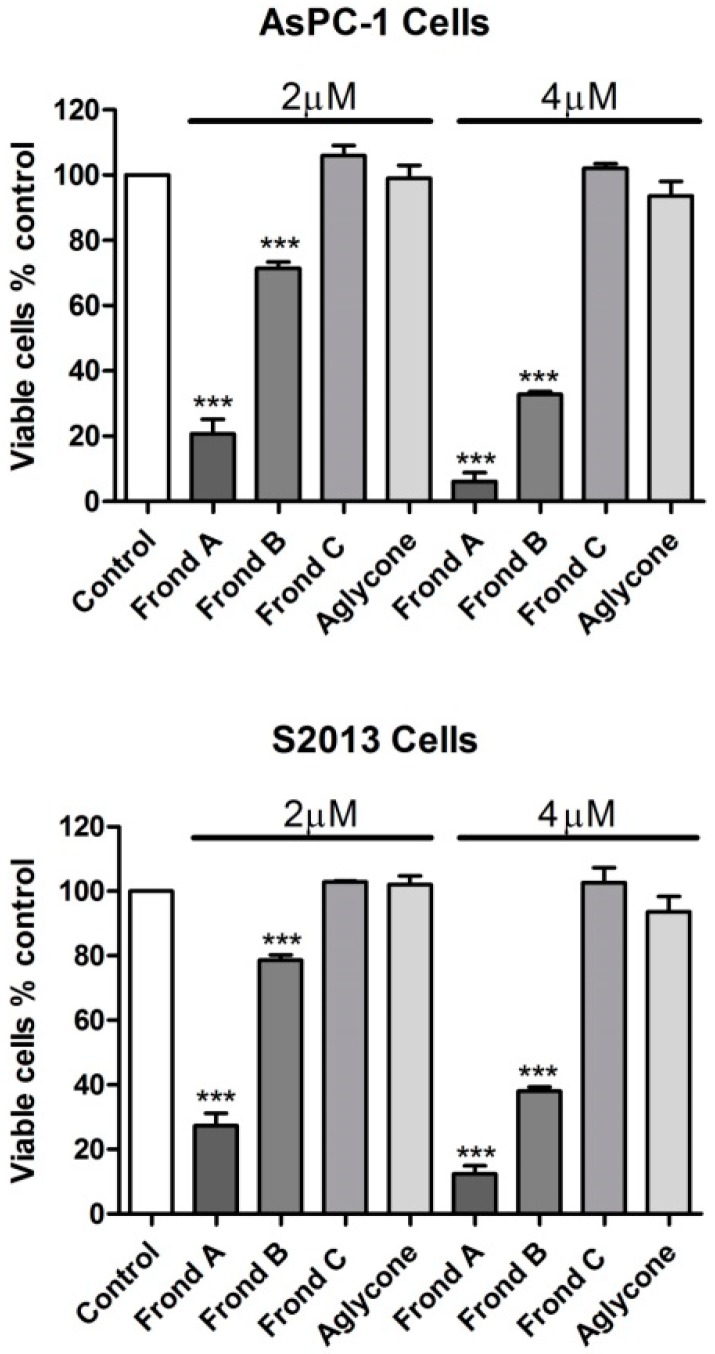
Effects of frondoside A, B and C and their aglycone on viability of AsPC-1 and S2013 human pancreatic cancer cells after 48 h of incubation. *** *p* < 0.001.

**Figure 3:**
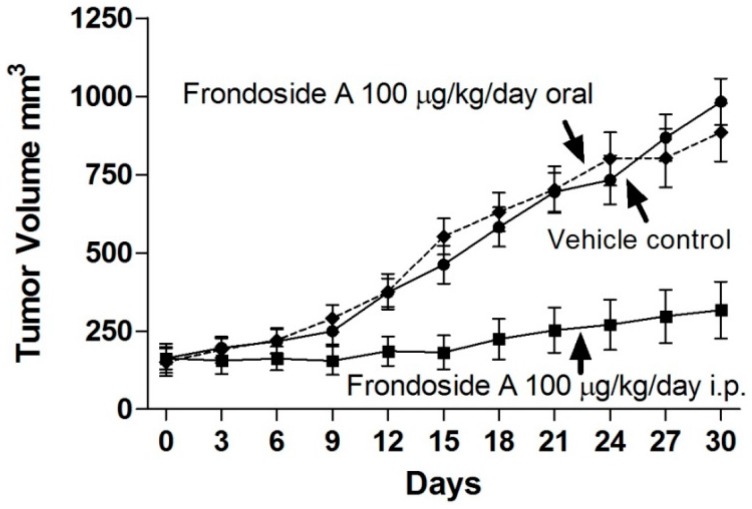
Effects of frondoside A administered either orally or intraperitoneally at a dose of 100 µg/kg/day on growth of AsPC-1 human pancreatic cancer xenografts in athymic mice over a 30-day period.

Both frondoside A and frondoside B markedly inhibited growth of human AsPC-1 and S2-013 cells (Figure 1). Frondoside A showed similar potency to that of previous reports in these cell lines, while frondoside B was less potent. The different frondosides and their aglycone were examined at concentrations of 2, 4, 6, 8, and 10 µM. Frondoside A at 2 µM inhibited cell growth by 70%–80% in AsPC-1 and S2013 cells at 48 h and by 90%–95% at 4 µM in both cell lines (All *p* < 0.0001, Figure 2). Frondoside B inhibited cell growth by around 20%–25% at 2 µM in the two cell lines and by about 60%–70% at 4 µM at 48 h (All *p* < 0.0001 except frondoside B at 2 µM: *p* < 0.05, Figure 3). Neither frondoside C nor the aglycone had a significant effect on cell viability at concentrations of 2 and 4 µM (Figure 2).

### 2.2. Comparison of Route of Administration of Frondoside A on Growth of AsPC-1 Xenografts in Athymic Mice

Frondoside A administered via an intraperitoneal route daily at 100 µg/kg/day substantially reduced growth of AsPC-1 xenografts in athymic mice over a 30 day period (Figure 3). In contrast, the same dose administered orally had no effect (Figure 3). When measured as the incremental area under the curve, tumor volume in the intraperitoneal frondoside A-treated group (AUC 1716 ± 2001) was markedly reduced compared with the control group (AUC 11, 184 ± 1812, *p* < 0.001), while oral frondoside A had no effect (AUC 11, 844 ± 2079).

### 2.3. Pharmacokinetics of Frondoside A

The assay was found to be suitable for measurement of frondoside A in both mouse and human plasma. Accuracy was 88%, within day coefficient of variation (CV) <8% for concentrations in the range of 25–250 ng/mL. The limit of detection (LOD) was 5 ng/mL. Results of the ultracentrifugation studies revealed 68%–80% binding of frondoside A to plasma proteins at concentrations between 250 and 100 ng/mL. With regard to stability, there was little change in concentrations of frondoside A in mouse or human plasma incubated at 37 °C for 1 h, but at 24 h 73% of the initial concentration (200 ng/mL) remained in human plasma while only 50% remained in mouse plasma. Protein binding was determined by ultracentrifugation. Pilot toxicity studies revealed no acute clinical signs of toxicity following single intravenous (i.v.), intraperitoneal (i.p.), or oral doses up to 300 µg/kg. Pharmacokinetic studies were carried out using intravenous (i.v.), intraperitoneal (i.p.), or oral dosing at 100 and 300 µg/kg. No adverse acute clinical signs were seen following any of these routes of administration. Plasma levels after i.v. dosing were readily measurable (700–800 ng/mL). Levels with i.p. dosing were much lower (~50 ng/mL) and levels after oral administration were near the limit of detection. The definitive study on pharmacokinetics was performed in CD_2_F_1_ mice with i.v. administration of frondoside A at 100 µg/kg.

**Figure 4:**
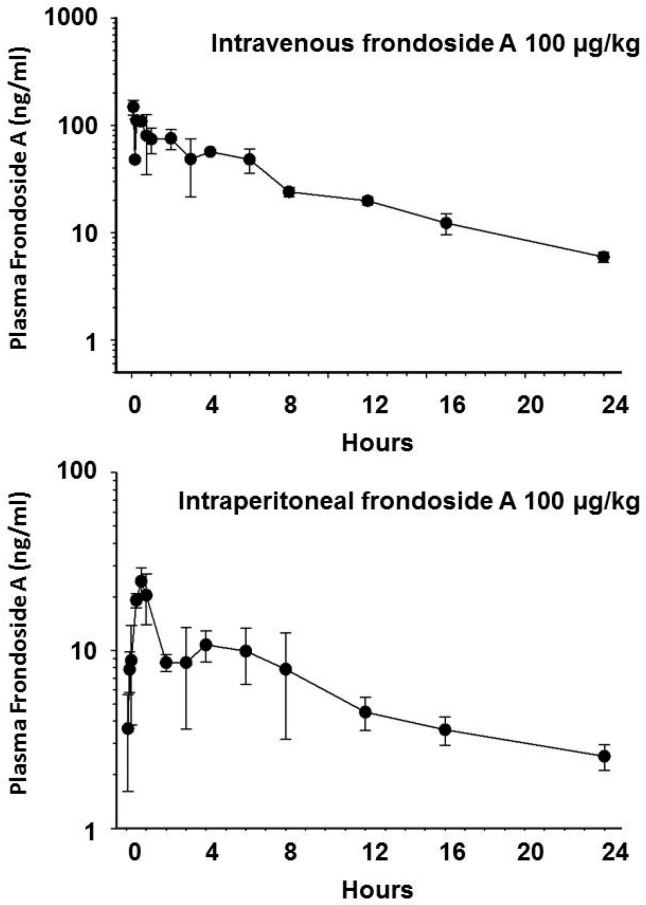
Plasma concentration of frondoside A *versus* time plot following administration of frondoside A at a dose of 100 µg/kg intravenously or intraperitoneally in CD2F1 mice. Each point represents the mean and SD of plasma concentration in five animals.

The pharmacokinetic parameters measured during the i.v. and i.p. experiments are shown in Table 1 and the concentration *versus* time plots are shown in Figure 4. The mean C_max_ following i.v. administration of frondoside A was 129 nM (172 ng/mL). The C_max_ following i.p. administration of frondoside A was 18.3 mM (24 ng/mL) at 45 min, which was approximately 7-fold lower than with i.v administration at the same dose (Figure 4).

The calculated bioavailability after i.p. administration was approximately 20%. Following i.v. dosing, plasma levels of frondoside A remained above 7.5 nM (10 ng/mL) for 17 h, while for i.p. dosing the plasma concentration remained above this level for only 4 h. Pilot studies with oral doses of 100 and 500 µg/kg demonstrated very low and variable concentrations of frondoside A in plasma that were near to or below the detection limit (5 ng/mL) of the assay. Pharmacokinetic parameters measured during the i.v. and i.p. experiments are shown in Table 1. Notably, the half-life of frondoside A administered intravenously was 8.5 h.

## 3. Discussion

In recent years, there has been considerable interest in sea cucumber-derived triterpenoid glycosides as anti-cancer agents. Frondoside A, from the Atlantic sea cucumber *Cucumaria frondosa*, has potent anti-cancer effects in cancers of the pancreas, lung, breast, prostate, and in leukemias, both *in vitro* and *in vivo* [4,5,6,7,8]. Frondoside A, causes cell cycle arrest, inhibits proliferation, and induces apoptosis [4,6,7]. In addition, frondoside A inhibits angiogenesis, invasion and metastases [5,6,7]. Frondoside A also potentiates the effects of other anti-cancer agents [9].

Frondoside A appears to be safe, with no apparent side effects, body weight, liver function and haematological parameters are not altered in animals that have received daily therapeutic doses of up to 10 or 100 µg/kg/day for one month [4,5,6,10]. The LD_50_ determined in previous studies was approximately 10 mg/kg, which is 100-fold greater than the high therapeutic dose used in previous preclinical experiments [10].

From the present studies it is clear that frondoside A has a more potent effect than frondoside B *in vitro*, while frondoside C had no effect at all in the concentration range tested. Similarly, the aglycone had no effect, while frondoside A inhibited proliferation with an IC_50_ of approximately 1 µM.

In the mouse xenograft experiment, intraperitoneal administration of frondoside A had a marked and highly significant inhibitory effect on tumor growth compared with vehicle control; tumor weights at the end of the experiment were less than 30% of the vehicle-treated controls. In contrast, orally administered frondoside A at the same daily dose had no significant effect on tumor size throughout the experiment or tumor weight at the end. These findings suggest that frondoside A is either not absorbed intact from the gut in any significant quantity or is rapidly metabolized by the animal’s digestive enzymes to inactive compounds. It is likely that the glycosyl groups are digested during passage through the gut and, as shown above, the aglycone has no tumor-inhibitory activity. This was corroborated by the pharmacokinetic studies that showed that plasma frondoside A levels after intravenous administration at a dose of 500 mg/kg were approximately 700–800 ng/mL, while they were approximately 100–120 ng/mL after intraperitoneal administration and were at or below the limit of detection of the assay (5 ng/mL) after oral administration. This data indicates that frondoside A is most effective when administered intravenously and is ineffective when administered through the oral route.

In summary, the additional sulphate group on frondoside B renders it less effective as an anticancer agent than frondoside A. The addition of two sulphate groups in frondoside C, or the removal of the glycoside moieties from the frondosides (to produce the aglycone) results in complete loss of activity. Frondoside A appears not to be absorbed intact from the gastrointestinal tract and is only effective as an anticancer agent when administered parenterally.

## 4. Materials and Methods

### 4.1. Isolation and Purification of Frondoside A, B, C and Their Aglycone

**Figure 5:**
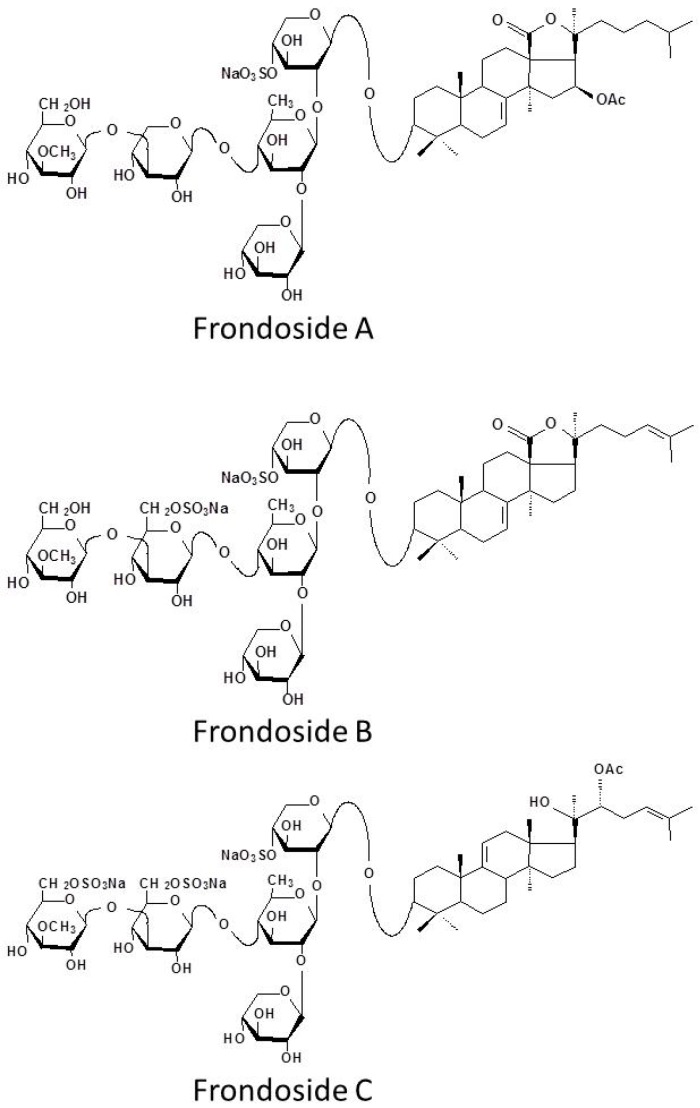
Structures of frondosides A, B, C (mono, di and tri-sulphated frondoside).

Frondosides (A, B and C) were isolated and purified as previously described [10]. For structures of these three compounds see Figure 5. A Biotage (Charlottesville, VA, USA) Si 40L 2632-2 flash column was used to separate mono-, di-, and trisulfated (frondoside A, B and C respectively) glycoside fractions using chloroform/ethanol/water (100:100:17, by volume) as a solvent system. For this, a part of crude glycoside fraction was dissolved in a minimal volume with the same solvent mixture and water by drops and loaded into the column. The column was then eluted with 0.5 L of the same solvent mixture with subsequent collections of 10-mL fractions. After completion of the frondoside A elution, the solvent mixture was changed to chloroform/ethanol/water (100:150:50 by volume). Eluting fractions were checked with thin-layer chromatography. The thin-layer chromatography solvent system was chloroform/ethanol/water (100:100:17 by volume). As a result, frondoside A (50 mg), a fraction of disulfated glycosides (136 mg) (frondoside B), and a fraction of trisulfated glycosides (171 mg) (frondoside C) were obtained. The aglycone was prepared by acid hydrolysis [12].

### 4.2. Cell Lines and Cell Cultures

The pancreatic cancer cell lines chosen for these studies, AsPC-1 and S2013 are both highly malignant and produce rapidly growing tumors in athymic mice. AsPC-1 is a poorly differentiated cell line derived from nude mouse xenograft initiated with cells from the ascites of a patient with pancreatic cancer (American Type Culture Collection (Manassas, VA, USA)) [13]. S2-013, a subclone of SUIT-2, is a well-differentiated cell line derived from a liver metastasis of human pancreatic cancer [14]. The S2-013 cells were cultured in Dulbecco’s Modified Eagles Medium (DMEM) and the AsPC-1 cells were grown in RPMI. Both media were supplemented with 10% fetal bovine serum, penicillin (100 units/mL), streptomycin (100 µg/mL) (Gibco, Grand Island, NY, USA) at 37 °C in humidified air with 5% CO_2_ for 24 h. Then cells were transferred into serum-free media after washing twice with PBS and incubated for 24 h in 24-well plates. Cells then were treated in fresh serum-free media with different concentrations of frondoside A, frondoside B, frondoside C and their aglycone and kept for incubation for 24 to 48 h. Cells were harvested by incubation in trypsin-EDTA solution for 10 min. Cells were then centrifuged at 2000× *g* for 2 min and cell pellets were suspended in fresh serum free media prior to measuring cell proliferation.

### 4.3. Cell Proliferation Assays

Cell proliferation was measured by counting the number of viable cells (Guava ViaCount) on Guava on an EasyCyte Plus cytometer (Millipore, Hayward, CA, USA).

### 4.4. Animals and Subcutaneous Tumor Cell Implantation

AsPC-1 cells were seeded into 75 cm^2^ flasks and cultured in a humidified atmosphere of 95% O_2_ and 5% CO_2_ at 37 °C, and media was changed every other day. The tumor cells were trypsinized, cell number counted (Guava EasyCytePlusCytometer) and then re-suspended in PBS.

For cancer xenografts 6–8 week old athymic nude mice were bred in the animal facility. Breeding stock was NMRI female nude mice (*nu*/*nu*, Charles River, Suizfeld, Germany). The mice were housed in micro-isolator cages in a filtered-air laminar flow isolation chamber and handled under aseptic conditions (EuroBioConcept, Paris, France). They were fed with autoclaved laboratory rodent food pellets and acclimatized to the facility for two weeks prior to tumor cell implantation. Animal weights were recorded every third day. The animal protocol was approved by the Institutional Animals Care and Use Committee and all procedures were conducted in accordance with Institutional Guidelines that are in compliance with Faculty of Medicine & Health Sciences, national and international laws and policies (EEC Council Directive 86/609, OJ L 358, 1; and NIH Guide for Care and Use of Laboratory Animals, NIH Publication No. 85–23).

Six million AsPC-1 human pancreatic cancer cells were injected into each flank of the athymic mice. Once tumors were established and had reached approximately 250 mm^3^, 7 days after injection, the mice were randomized into 3 groups with 6 mice per group: Group I (Control) received daily i.p. injections of vehicle (100 µL 0.2% DMSO); Group II (frondoside A) received daily i.p. injections of frondoside A 100 µg/kg/day (4 µL/g of 25 µg/mL 0.2% DMSO); Group III (frondoside A) received daily oral dose of frondoside A 100 µg/kg/day (4 µL/g of 25 µg/mL 0.2% DMSO);. Animal weight and tumor size were recorded every third day. The formula for calculating tumor volume was: (length) × (width) × (length + width/2) × 0.526 = volume [15]. After 30 days of treatment the animals were euthanized and the tumors were carefully dissected and tumor weights measured. Tissues were fixed in 4% formaldehyde, which was replaced with 70% ethanol after 24 h. Tissues were then paraffin embedded according to histology standard protocols.

### 4.5. Pharmacokinetics of Frondoside A

An LC-MS/MS method was developed and validated for the measurement of frondoside A in mouse and human plasma. Frondoside A was extracted from plasma as follows. A 100 mg C18-E extraction cartridge (Phemonenex, Torrance, CA, USA) was prepared by washing with 1 mL methanol followed by 1 mL water. Samples (100 µL plasma + 400 µL water) were then loaded onto the cartridge followed by washing with 1 mL water. The wash was discarded and frondoside eluted using 3 mL of a solution containing methanol:acetonitrile:acetic acid (60:40:0.1, V:V:V). The eluent was dried at 40 °C and reconstituted with 100 µL of 1:1 methanol: 10 mM ammonium acetate. Samples were vortexed for 30 s and then transferred to a clean vial for injection onto the LC/MS/MS.

Chromatography was carried out by injection of 25 µL aliquots of extracted plasma samples on a Phenomenex Luna C5 column (50 × 2 mm) using an elution gradient with 10 mM ammonium acetate, pH 8.5 and methanol, at a flow rate of 300 µL/min at 60 °C. The column was connected to a MicroMass Quatro Ultima tandem mass spectrometer (Waters Corp., Milford, MA, USA) operated in electrospray negative mode. Compounds were detected at *m*/*z* 1331.5 → 1331.5, with bardoxolone methyl ester (CDDO) as an internal standard (MS/MS at *m*/*z* 490.4 → 416.2).

Pilot studies were conducted in CD_2_F_1_ mice to determine the optimal doses for the subsequent pharmacokinetic studies. The definitive studies on pharmacokinetics were performed in CD_2_F_1_ mice with intravenous (i.v.) or intraperitoneal (i.p.) administration of frondoside A at 100 µg/kg. Blood was collected from 5 animals per time point, the plasma collected, solid-phase extracted and frondoside A levels measured using the LC-MS/MS methods described above. Concentration-time data were fitted by non-linear least-squares regression.

### 4.6. Statistical Analysis

All data were analyzed using analysis of variance (ANOVA) with Bonferroni’s or Dunnett’s corrections for multiple comparisons as appropriate. The growth curves for tumor growth *in vivo* were analyzed as incremental area under the curve for statistical purposes. Differences were considered statistically significant when *p* ≤ 0.05. Graphs were created using either GraphPad Prism or SigmaPlot software.

## 5. Conclusions

Frondoside A is a more effective anticancer agent than frondoside B. Frondoside C and the aglycone have no anticancer activity. Frondoside A appears not to be absorbed intact from the gastrointestinal tract in significant amounts and is only effective as an anticancer agent when administered parenterally. Frondoside A may be valuable in the treatment of cancer.

## Figures and Tables

**Table 1:** Pharmacokinetics of frondoside A following bolus injection of 100 µg/kg in 0.7% DMSO in saline, either intraperitoneally or intravenously, in 10 male CD_2_F_1_ mice.

Parameter	IP Bolus	IV Bolus
Area under curve (AUC) µg/L × min	9984	47,220
Total body clearance (Cl_tb_) mL/min/m^2^	127	6.35
Maximum plasma concentration (Cp_max_) nM	18.3	129
Bioavailability (%)	20	100
Apparent volume of distribution (L/m^2^)	28	-
Volume of distribution (L/m^2^)	-	0.87
Half-life γ (T_½_ γ) minutes	840	510
Half-life α (T_½_ α: distribution phase) minutes	-	2
Half-life β (T_½_ β elimination phase) minutes	-	158

## Abbreviations

The following abbreviations are used in this manuscript:

AUC: Area under curve
Cl_tb_: Total body clearance
Cp_max_: Maximum plasma concentration
CV: Coefficient of variation
DMSO: Dimethyl sulphoxide
LOD: Limit of detection
V_D_: Volume of distribution
